# Clinical presentation and management of COVID‐19

**DOI:** 10.5694/mja2.50698

**Published:** 2020-07-17

**Authors:** Irani Thevarajan, Kirsty L Buising, Benjamin C Cowie

**Affiliations:** ^1^ Victorian Infectious Diseases Service Royal Melbourne Hospital Melbourne VIC; ^2^ University of Melbourne Melbourne VIC; ^3^ WHO Collaborating Centre for Viral Hepatitis Doherty Institute Melbourne VIC

**Keywords:** Clinical decision‐making, Respiratory tract infections, Epidemics, COVID‐19

## Abstract

- The rapid spread of severe acute respiratory syndrome coronavirus 2 led to the declaration of a global pandemic within 3 months of its emergence.
- The majority of patients presenting with coronavirus disease 2019 (COVID‐19) experience a mild illness that can usually be managed in the community. Patients require careful monitoring and early referral to hospital if any signs of clinical deterioration occur.
- Increased age and the presence of comorbidities are associated with more severe disease and poorer outcomes.
- Treatment for COVID‐19 is currently predominantly supportive care, focused on appropriate management of respiratory dysfunction.
- Clinical evidence is emerging for some specific therapies (including antiviral and immune‐modulating agents). Investigational therapies for COVID‐19 should be used in the context of approved randomised controlled trials.
- Australian clinicians need to be able to recognise, diagnose, manage and appropriately refer patients affected by COVID‐19, with thousands of cases likely to present over the coming years.

In December 2019, a novel coronavirus emerged in Wuhan, Hubei Province, China, leading to a global pandemic. The virus, named severe acute respiratory syndrome coronavirus 2 (SARS‐CoV‐2), causes a clinical syndrome termed coronavirus disease 2019 (COVID‐19).

The first reports of an undiagnosed pneumonia in Wuhan on 8 December 2019 were followed by an alert from China to the World Health Organization (WHO) about a cluster of pneumonia cases on 30 December. Isolation of a novel coronavirus occurred on 3 January 2020. On 30 January, the WHO declared a public health emergency of international concern, and a pandemic was declared on 12 March 2020.

## Clinical presentation

Similar to other coronaviruses, SARS‐CoV‐2 is predominantly spread by respiratory droplets, although spread by contact with contaminated fomites also occurs, as does transmission by aerosols in certain circumstances.^1^

Based on the experience in China, the typical incubation period of COVID‐19 infection has been estimated to be a median of 5.1 days (95% CI, 4.5–5.8 days), with 97.5% of those who develop symptoms doing so within 11 days of exposure (95% CI, 8.2–15.6 days). This has informed the use of a 14‐day time period for quarantining potentially exposed individuals in an effort to limit onward spread.^2^

The recognition of asymptomatic infection has been an area of intense interest in understanding the epidemiology of COVID‐19. The ratio of asymptomatic to symptomatic infection is currently uncertain. Cross‐sectional studies have reported asymptomatic infection in women attending a maternity service in New York (33 of 215 infected, 88% asymptomatic)^3^ and in general population testing in Iceland (87 of 10 797 infected, 41% asymptomatic).^4^ In such cross‐sectional studies, a proportion of those who were asymptomatic at the time of testing may in fact have been in the pre‐symptomatic phase of infection. In a study conducted in a nursing home in the United States, 48 of 76 residents tested positive, with 27 (56%) being asymptomatic at the time of testing. However, 24 (89%) of these individuals went on to develop symptoms at a median of 4 days (interquartile range [IQR], 3–5 days) after the positive test result.^5^

Symptomatic COVID‐19 infection usually presents as a respiratory syndrome, most commonly with fever and cough.^6^, ^7^

Fever has been reported in up to 99% of people at some time during the course of their illness, but importantly in one cohort, it was reported to be present at the time of hospital presentation in only 44% of patients, and at some time during the hospital admission in 89%.^8^ Other common symptoms are cough, dyspnoea, fatigue, anorexia, anosmia, myalgia and sometimes confusion. Diarrhoea may occur in up to 10% of patients.^9^ Symptoms reported less frequently (< 5% of cases) include sore throat, rhinorrhoea, headache, chest pain, dizziness, abdominal pain and nausea.^6^, ^7^

Around 80% of COVID‐19 infections present as a mild respiratory illness in a patient who is ambulatory and can generally be managed outside the hospital. Around 15% typically need hospital care (usually for moderate to severe pneumonia), and another 5% have critical illness requiring more intensive supports.^10^

Of those who require hospitalisation, the median time from first symptoms to onset of dyspnoea is 5 days (IQR, 1–10 days), the median time to hospital admission is 7 days (IQR, 4–8 days), and in those who develop more severe manifestations, the median time to acute respiratory distress syndrome is 8 days (IQR, 6–12 days).^6^ About a quarter of patients who are hospitalised may need transfer to the intensive care unit (ICU) for the management of complications such as hypoxaemic respiratory failure or hypotension requiring vasopressor support.^11^

At presentation to hospital, the most common laboratory feature of COVID‐19 infection is lymphopenia (reported in 70.3% of cases).^6^ Radiological imaging may reveal a clear chest, unilateral or bilateral consolidation, or ground glass opacity.

## Diagnosis

Nasopharyngeal specimens, deep nasal swabs, throat swabs or lower respiratory samples (eg, sputum) sent for molecular detection of SARS‐CoV‐2 by polymerase chain reaction (PCR) are currently the best means of specific diagnosis of COVID‐19 in Australia. Faecal samples may also be PCR positive for COVID‐19 but the role of the oral–faecal route for transmission remains unclear.^12^ Patients with more severe disease tend to have higher viral loads in respiratory samples. Mild cases have been shown to clear the virus earlier, with over 90% returning negative PCR test results by day 10 compared with severe cases who more often remain positive beyond day 10.^13^ Viral loads appear to be highest early in the illness. Prolonged viral shedding after the onset of symptoms has been described.^14^ The virus has also been detected by PCR in asymptomatic patients with comparable viral loads to those still symptomatic.^15^

## Assessment

Patients with suspected or confirmed COVID‐19 should be assessed for features of severe disease and risk factors for progression to severe disease. This assists in determining whether a patient can safely be managed in the community or requires referral and admission to a health care facility able to provide acute inpatient care. Current data suggest that older patients and those with comorbidities have increased risk of progression to severe disease and mortality. In a large surveillance report from China including over 44 000 confirmed cases of COVID‐19, the case fatality rate was < 0.5% for patients aged < 50 years, but rose to 8.0% for those in their 70s, and 14.8% in those aged > 80 years.^16^ While these surveillance‐based case fatality rates are possibly overestimates, being influenced by under‐recognition of lower severity cases, the impact of increasing age and the presence of comorbidities on risk of severe and fatal illness should be recognised,^8^ and such patients should generally be offered more careful monitoring.

Clinical features that have been identified more often in COVID‐19 infected patients who have had a fatal outcome compared with those who survive are: dyspnoea at presentation (70.6% *v* 24.7%; *P* < 0.001); lower initial oxygen saturation (median oxygen saturation, 85% [IQR, 75–91%] *v* 97% [IQR, 95–98%]; *P* < 0.001); and higher total white blood cell count but lower lymphocyte count at presentation accompanied by a lower lymphocyte count, expressed as a lower lymphocyte percentage (median, 7.1% [IQR, 4.5–12.7%] *v* 23.5% [IQR, 15.3–31.3%]; *P* < 0.001).^17^ In developing a predictive model, Chinese researchers found four factors independently associated with disease progression during hospitalisation in 208 consecutive patients: presence of comorbidity, age > 60 years, lymphocyte count < 1.0 × 10^9^/L, and elevated lactate dehydrogenase levels.^18^

A propensity for deterioration in the second week of illness has been recognised in some cohorts of patients, typically 5–10 days after the onset of symptoms.^19^ All patients should be warned about symptoms of concern (such as increasing breathlessness), and early referral for hospital admission should be suggested for any patient with signs of clinical deterioration. Individual circumstances need to be considered when determining the ideal monitoring strategy and site of care for each patient (Box).

Box 1 **Assessing disease severity and consideration for setting of care for patients diagnosed with COVID‐19**

**Table:** 

Disease severity	Clinical features	Setting of care
Mild illness/lower risk of progression to severe disease	Mild upper respiratory symptoms (eg, cough, sore throat, myalgia, fatigue) AND Age < 60 years AND No major comorbidities	Ideally manage out of hospital (eg, at home or in a step‐down facility), unless symptoms progress to lower tract symptoms such as dyspnoea (see below)
Moderate illness/intermediate risk of progression to severe disease	Stable patient presenting with respiratory and systemic symptoms or signs: severe asthenia, prostration, fever > 38°C or productive cough clinical or radiological signs of lung involvement but no signs of severe pneumonia no clinical or laboratory indicators of clinical severity or respiratory impairment AND No major comorbidities	If patient amenable to community level management, careful monitoring into second week of illness is recommended AND Early referral for hospital admission if any evidence of clinical deterioration
Severe illness	Patient meeting any of the following criteria: dyspnoea at rest or minimal activity (talking, sitting) SpO_2_ on room air < 92% respiratory rate > 22 breaths/min haemodynamically unstable (systolic blood pressure < 100 mmHg) extensive chest x‐ray infiltrate, or rapid worsening from baseline	Assessment for hospital admission
Clinical deterioration and at risk for critical illness	Worsening respiratory state as determined by any of the following criteria: new requirement for oxygen support to maintain SpO_2_ > 92% escalating oxygen requirements increasing respiratory rate or work of breathing oxygen requirement > 6 L/min systolic blood pressure decline not responding to judicious fluid therapy impairment of consciousness other organ failure	Early referral to intensive care unit if goals of care include intensive care unit management

Adapted from World Health Organization interim guidance,^21^ Australasian Society for Infectious Diseases interim guidelines,^20^ and National COVID‐19 Clinical Evidence Taskforce living guidelines.^19^

## General management

It is critically important to ensure optimal infection prevention from the time a patient with suspected COVID‐19 is first assessed until their infection is resolved, irrespective of the site of care. This can present particular challenges for health care staff, who must learn to use personal protective equipment safely, and for patients and their loved ones who must manage the difficulties associated with isolation.

Patients with mild disease (about 80%)^10^ can often be managed in the community if they are able to self‐isolate. They must also be capable of monitoring their own condition, be aware of which symptoms should prompt medical review, and be able to escalate any concerns.^19^, ^20^, ^21^ For some patients, a more proactive program of monitoring by phone or telehealth or in‐person monitoring (eg, hospital in the home, regular review by general practitioner, or hospital admission) may be required. Strategies for care should be individualised to suit patient circumstances. Patients whose home environment is not conducive to safe management, or which is unacceptable from an infection prevention perspective, may require admission either to hospital or to alternative safe accommodation. Discussion with public health authorities is essential to ensure that appropriate isolation and follow‐up mechanisms are in place. In the face of high health care demand during the peak of a pandemic, safe management of low risk patients in the community will likely be essential to preserve hospital capacity for the more severely ill.

Patients with moderate or severe illness will generally require admission to hospital. This includes those who are dyspnoeic on minor exertion, tachypnoeic at rest (respiratory rate > 22 breaths/min), hypoxaemic (pulse oximetry [SpO_2_] < 94% on room air), hypotensive (systolic blood pressure < 100 mmHg), have an acutely altered mental state, or who have extensive pulmonary infiltrates evident on chest imaging.^19^, ^20^, ^21^

Severe illness, indicated by, among other features, a respiratory rate > 30 breaths/min, SpO_2_ < 92% on room air^19^, ^21^ or sustained hypotension, warrants urgent hospitalisation and consideration of the need for intensive care if suitable for a given patient.

## Respiratory management

Supplemental oxygen should be administered for patients with SpO_2_ < 92%.^19^, ^20^ Once stabilised, the target SpO_2_ range is usually 92–96%. The target will be lower in those with chronic hypercapnoeic respiratory failure (eg, 88–92%).^19^, ^20^, ^21^

Manoeuvres to improve gas exchange should be implemented, such as positioning patients appropriately in bed (on either side with regular turning), elevating the bed head to 30 degrees, encouraging deep breathing every hour while awake, sitting patients out of bed every day when possible, and mobilising when able. For mechanically ventilated patients with persistent hypoxaemia, prone positioning may be effective.^19^, ^22^

In the setting of progressive hypoxaemia despite low or moderate flow oxygen (via nasal prongs or Hudson mask), high flow oxygen can be considered. Whether high flow oxygen devices (> 10 mL/min) are potentially aerosol‐generating is being studied, but current guidelines^1^, ^23^ advise that airborne precautions be taken by staff (personal protective equipment including N95/P2 masks) and single rooms where possible.

There are emerging views that the respiratory dysfunction observed in COVID‐19 infections is not uniform.^22^ Initial recommendations have focused on consideration of early intubation and mechanical ventilation for patients with acute respiratory distress syndrome due to COVID‐19.^1^, ^19^, ^20^, ^21^ Experience from a multicentre Italian COVID‐19 patient cohort suggests that non‐invasive ventilation such as continuous positive airways pressure and bilevel positive airways pressure may also have a role both within and outside ICUs.^24^ These non‐invasive ventilation devices are clearly aerosol‐generating and as such should only be used with appropriate precautions in place.^1^, ^23^ Advice from experts in respiratory medicine or critical care should be sought.

## Other management considerations

Empirical antibiotic therapy for bacterial pneumonia should be considered in patients whose illness is severe, where there is evidence of sepsis or septic shock, or where the patient is clinically deteriorating.^19^, ^20^ Empirical treatment for influenza with a neuraminidase inhibitor should be considered for patients with severe pneumonia (guided by local epidemiology) until influenza PCR results are available.^20^, ^21^ Empirical antibiotics are not recommended for patients with mild or moderate pneumonia unless there is additional clinical evidence to suggest bacterial infection. De‐escalation of empirical antimicrobial therapy should be undertaken as appropriate, guided by microbiology results (where available) and clinical judgement.^21^

Hypovolaemia may be contributed to by reduced oral intake and increased losses, but management requires cautious administration of intravenous fluids with regular assessments given the risk of exacerbating pulmonary oedema in the setting of acute respiratory distress syndrome^19^, ^22^ and given the possibility of underlying cardiac injury.^25^

A range of possible complications related to SARS‐CoV‐2 infection have been reported and their incidence is being monitored. These include thromboembolic events in the lungs^22^ and cerebrovascular system,^26^ Prophylaxis with anticoagulants for adults with moderate, severe or critical COVID‐19 infection is generally recommended, unless there are contraindications.^19^, ^21^ Acute cardiac injury presenting with electrocardiogram changes, arrhythmias, left ventricular dysfunction, cardiomyopathy and congestive cardiac failure have also been described, and assessment of baseline electrocardiogram is suggested for patients with moderate or severe COVID‐19 illness.^25^, ^27^

There is considerable interest in monitoring large patient cohorts and conducting analysis of linked datasets at a population level to establish whether there are any rare or longer term complications or associations of COVID‐19 with other medical conditions. Given the very recent emergence of SARS‐CoV‐2, data are currently limited but it is likely that information will emerge in coming months from populations that have experienced a high attack rate. An example of a rare condition with potential association is paediatric inflammatory multisystem syndrome temporally associated with SARS‐CoV‐2, presenting as hyperinflammatory shock with features similar to atypical Kawasaki disease.^28^ Similarly, there is interest in monitoring long term incidence of cardiovascular complications, thromboembolic disease, chronic respiratory dysfunction, renal or neurological disorders, and post‐infectious inflammatory syndromes after COVID‐19, in addition to inspection of large datasets for complications that are as yet unsuspected.

## Specific therapies

A range of pharmacotherapies have been proposed as possible treatments for COVID‐19. Early evidence of clinical benefit for some agents has emerged. The WHO interim guidance on the clinical management of COVID‐19^21^ asserts that investigational therapeutics should be used only in approved randomised controlled trials. This position is endorsed by the Australasian Society for Infectious Diseases interim guidelines for the clinical management of COVID‐19 in adults,^20^ and the Australian guidelines for the clinical care of people with COVID‐19,^19^ which state that even where conditional recommendations for use of disease modifying agents are made, whenever possible these should be administered in the context of randomised trials with appropriate ethical approval.

The understandable interest in evaluating potential treatments has led to a large number of clinical trials being registered globally; by late April 2020, over 1100 clinical studies were registered, including over 500 randomised controlled trials.^29^

### Antimicrobials

#### Lopinavir–ritonavir

Lopinavir–ritonavir, a combined antiretroviral agent, was proposed as a potential treatment for severe acute respiratory syndrome in 2003, based on apparent reductions in mortality in preliminary research in Hong Kong.^30^ Given its hypothesised role, five of the first 18 patients diagnosed with COVID‐19 in Singapore were administered this agent.^31^

On 18 March 2020, a randomised controlled open label trial of lopinavir–ritonavir in 199 hospitalised adults with COVID‐19 in China was published.^32^ No benefit was observed in participants treated with the antiviral compared with controls. Nearly 14% of those receiving lopinavir–ritonavir were unable to complete 14 days of treatments owing to adverse events.

#### Chloroquine and hydroxychloroquine

Chloroquine and hydroxychloroquine are antimalarial agents which also have immunomodulatory properties that led to established indications for use in the treatment of rheumatological conditions. Potential adverse effects include retinal toxicity, QT interval prolongation and other cardiological and dermatological effects.

In early February 2020, chloroquine was reported to inhibit SARS‐CoV‐2 replication in vitro.^33^ By mid‐February, treatment of COVID‐19 with chloroquine was being described as a “breakthrough”: a published letter stated that the results of treatment in over 100 patients in China had demonstrated that chloroquine was “superior to the control treatment”, but no data were provided.^34^ A small French open label non‐randomised clinical trial examining hydroxychloroquine with or without azithromycin suggested a significant viral load reduction in those receiving therapy;^35^ however, concerns have been raised about the design and analysis of the study.^36^

Despite the lack of clinical evidence from randomised clinical trials, several institutional and local guidelines, and notable public figures, have supported the potential use of chloroquine or hydroxychloroquine for the treatment of COVID‐19.^37^, ^38^

However, given the current lack of evidence of clinical benefit and reports of significant limitations of supply of hydroxychloroquine for patients with rheumatological conditions, in March 2020, the Pharmaceutical Society of Australia and the Australasian Society for Infectious Diseases called for immediate cessation of prescribing and dispensing of hydroxychloroquine for indications relating to COVID‐19, outside use in approved clinical trials.^39^, ^40^

On 5 June 2020, the chief investigators on the RECOVERY trial (comprising over 11 500 patients enrolled from hospitals across the United Kingdom) issued a press release stating that no beneficial effect of hydroxychloroquine had been observed.^41^ No difference in 28‐day mortality, duration of admission, or other outcomes were observed between the 1542 patients randomised to hydroxychloroquine and the 3132 patients randomised to usual care. Further details regarding this analysis are awaited.

#### Remdesivir

In January 2020, the first patient diagnosed with COVID‐19 in the US received the investigational nucleotide prodrug remdesivir, supplied on a compassionate basis.^42^ Developed as a potential therapy for Ebola, there is in vitro evidence that remdesivir inhibits replication of coronaviruses, including Middle East respiratory syndrome coronavirus and SARS‐CoV‐2.^33^, ^43^ By late March 2020, four clinical trials to assess the efficacy of remdesivir against COVID‐19 had commenced in the US and two were registered in China.^44^ On 29 April, results of the first randomised clinical trial conducted in China were published;^45^ while this found no clinical benefit of remdesivir, the trial was underpowered (237 participants) owing to the success of public health measures in controlling COVID‐19 in China. The authors noted a non‐significant numerical reduction in time to clinical improvement in patients commencing treatment earlier in the course of illness.

On 27 May 2020, the first positive results of a randomised double‐blind controlled trial of a treatment for COVID‐19 were published.^46^ This international multicentre study reported the preliminary results of 1059 hospitalised patients who received up to 10 days of remdesivir or placebo. Those receiving remdesivir had a significantly shorter median recovery time of 11 days compared with 15 days for those receiving placebo (rate ratio for recovery, 1.32; 95% CI, 1.12–1.55; *P* < 0.001); no significant difference in mortality was found. Benefit was reported for the group requiring oxygen but not yet requiring invasive or non‐invasive ventilatory support. This new evidence has led Australian national guidelines to adopt a conditional recommendation for use of remdesivir outside of a trial setting where necessary.^19^

#### Combination therapy with interferon beta‐1b, lopinavir–ritonavir and ribavirin

In May 2020, a randomised trial in Hong Kong reported results of a comparison of lopinavir–ritonavir alone (*n* = 24) with a combination of lopinavir–ritonavir, ribavirin and subcutaneous interferon beta‐1b (*n* = 52).^47^ The combination group experienced a faster median time to viral clearance (7 days *v* 13 days; *P* < 0.0001) and shorter median length of hospital stay (8 days *v* 15 days; *P* = 0.0030) if the combination was commenced in the first 7 days from symptom onset. Importantly, the cohort of patients studied was not particularly unwell, with very few requiring ICU support and no deaths in the group.

### Immunomodulatory treatments

#### Corticosteroids

Interim guidance from the WHO states that corticosteroids should not be used in routine treatment of COVID‐19.^21^ This is based on systematic reviews in the context of severe acute respiratory syndrome and Middle East respiratory syndrome which showed lack of effectiveness, and possible harm.^48^

In a study of 138 hospitalised patients with COVID‐19 in Wuhan,^49^ 72.2% of ICU patients and 35.3% of non‐ICU patients received glucocorticoid therapy. The authors commented that while the dose of methylprednisolone varied depending on disease severity, no effective outcomes were observed.

However, on 22 June 2020, a preliminary report regarding interim findings from the UK RECOVERY trial suggested that low dose dexamethasone (6 mg daily orally or intravenous for 10 days) may substantially reduce mortality in hospitalised patients with COVID‐19 who received supplemental oxygen or mechanical ventilation.^50^ In comparing 2104 patients randomised to receive dexamethasone with 4321 randomised to receive usual care, dexamethasone was found to reduce mortality by 35% (rate ratio, 0.65; 95% CI, 0.51–0.82; *P* < 0.001) among ventilated patients, and for those receiving oxygen without mechanical ventilation, mortality was reduced by 20% (rate ratio, 0.80; 95% CI, 0.70–0.92; *P* = 0.002). No benefit of dexamethasone was observed among hospitalised patients who did not require respiratory support. While peer review and formal publication of this analysis is awaited, it is likely that these findings will be reflected in national and international guidelines.

#### Interleukin 6 antagonists

Tocilizumab is a humanised monoclonal antibody which binds to interleukin 6 (IL‐6) receptors, resulting in reduced immune activation and inflammation. It is licensed in Australia for use in autoimmune conditions including rheumatoid arthritis and giant cell arteritis. In addition to complications of immunosuppression including serious infections, adverse effects include hepatotoxicity and gastrointestinal complications. The theory behind use of tocilizumab or other agents that target the IL‐6 pathway (eg, sarilumab) in the context of COVID‐19 is that part of the pathogenesis in some patients may be attributable to an acute inflammatory syndrome or cytokine storm, which is associated with elevated IL‐6 levels. Clinical trials of these agents are currently underway.^44^

#### Other agents

Numerous immunomodulatory agents have been proposed as potential adjunctive treatments for COVID‐19, with a range of different immunological targets including other inflammatory cytokines. These include anakinra (an IL‐1 receptor antagonist), bevacizumab (an antivascular endothelial growth factor agent), and eculizumab (which inhibits terminal complement and prevents formation of the membrane attack complex).^44^, ^51^ While clinical trials are underway overseas for several proposed agents, no data exist to support their use at this time.^44^

### Passive immunotherapy

A preliminary, uncontrolled case series of five critically ill Chinese patients with COVID‐19 who received convalescent plasma containing high SARS‐CoV‐2‐specific antibody titres was published on 27 March 2020.^52^ While improvement in clinical status was reported following this intervention, the small sample size and uncontrolled nature of the study precludes drawing any conclusions regarding the efficacy of this intervention. Once again, further research is needed.

## Holistic care

A global pandemic causes understandable fear and anxiety for many people in the community. For those at particular risk of worse outcomes of infection — older people and those with significant pre‐existing illness or multiple comorbidities — COVID‐19 represents a particular threat. In addition, the health care workforce is under substantial strain and faces a potentially overwhelming challenge in delivering care to patients. Ensuring emotional care for the most vulnerable and those experiencing high levels of stress will be a fundamental determinant of the resilience of our society during this challenge.

For vulnerable and frail patients at particular risk of poor outcomes, it is important to provide personalised care and to develop an understanding of each individual's perspectives and preferences for health management. Involving caregivers and family members in decision making and establishing goals of care is necessary.^21^ Discussing goals of care early and, where appropriate, assisting patients to make advance care directives or resuscitation plans early in illness (or before infection) may provide substantial peace of mind and allow families to face the pandemic openly and with unity as they support vulnerable loved ones.

It is essential to ensure that all patients receive the best standard of care irrespective of the setting in which the care is delivered, or of the existence of any proposed limitations to life‐extending interventions. Under no circumstances should the best possible symptom control and compassionate, individualised care be denied any patient affected by COVID‐19.

## Conclusion

SARS‐CoV‐2 has caused a global pandemic with a profound public health impact, changing the daily lives of billions of people. It has exposed weaknesses in even strong and well resourced health systems internationally, and the economic impact alone will be staggering.

However, never before has the global community had the tools currently available to address a pandemic threat. A strong commitment to social and public health strategies and communicable disease control will ensure our health system retains the capacity to address COVID‐19, including sufficient hospital and intensive care resources to care for those with severe illness.

Biomedical innovations such as new and rapid point‐of‐care diagnostics, effective specific treatments and preventive vaccines are very high priorities which are rightly attracting substantial attention and funding. In the interim, high quality, evidence‐based clinical care — scaled up to face the pandemic challenge — together with robust public health interventions will save the lives of thousands in Australia, and millions globally.

## Competing interests

No relevant disclosures.

## Provenance

Commissioned; externally peer reviewed.
